# Editorial: The Extracellular Environment in Controlling Neuronal Migration During Neocortical Development

**DOI:** 10.3389/fcell.2021.673825

**Published:** 2021-04-29

**Authors:** Yuki Hirota, Chiaki Ohtaka-Maruyama, Victor Borrell

**Affiliations:** ^1^Department of Anatomy, Keio University School of Medicine, Tokyo, Japan; ^2^Neural Network Project, Department of Brain Development and Neural Regeneration, Tokyo Metropolitan Institute of Medical Science, Tokyo, Japan; ^3^Instituto de Neurociencias, Consejo Superior de Investigaciones Científicas and Universidad Miguel Hernández, Sant Joan d'Alacant, Spain

**Keywords:** cerebral cortex, development, neuronal migration, extracellular environment, evolution

In the developing brain, new neurons are generated at specific locations before migrating to their final destination, where they perform their adult functions. The cerebral cortex comprises neurons born in different regions and at different developmental times, but these are eventually organized into six layers according to their birth date. During this process, excitatory neurons undergo dynamic changes in their morphology and migration modes, including multipolar migration, locomotion, and terminal translocation. During development, neuronal migration impairment causes abnormal organization of the neocortex, resulting in various functional disorders such as epilepsy and neurological disability. Understanding the precise mechanisms underlying neuronal migration is a fundamental basis for understanding both neocortical development in the healthy brain and pathophysiological mechanisms underlying developmental neurological disorders.

During migration, neurons sense and respond to a broad range of extracellular environmental signals, including biochemical and mechanical cues, which serve as scaffolds to guide and support them. Genetic and molecular analyses have revealed signaling networks that precisely control the biochemical cellular microenvironment during neocortical development, including extracellular matrix (ECM) proteins, cell surface proteins, and gradients of diffusible guidance cues. Recent studies using live imaging and physical measurement techniques, such as atomic force microscopy (AFM), have revealed the essential role of the mechanical environment, including tissue stiffness, dynamic forces, and scaffolds, in neuronal migration. This Research Topic aims to highlight the critical relevance of the extracellular environment to various modes of neuronal migration in the mammalian brain under both physiological and pathological conditions.

Radial glial (RG) cells lining the ventricular surface function as primary neural progenitor cells, generating various neurons and glial cells. RG cells also serve as a physical scaffold supporting the radial migration of newborn neurons. In this Research Topic, Ferent et al. review the extracellular factors controlling the formation and maintenance of RG scaffolding. The first step of neuronal migration is cell delamination, in which neuronally differentiating cells generated from apical RG cells retract their apical processes and depart from the ventricular surface. Kawaguchi reviews the cellular and molecular mechanisms that regulate this process.

Cell adhesion molecules located on the cell surface play essential roles in the interaction between migrating neurons and their environment. The review by Martinez-Garay summarizes the function of cadherins during cortical migration, mainly focusing on CDH2, which plays critical roles in multiple steps of migration, including the apical delamination of newborn neurons, acquisition of polarity in the intermediate zone, RG-guided locomotion, and terminal soma translocation. More specifically, Peregrina and del Toro focus on reviewing the functions of the FLRT family of cell adhesion proteins to control the radial migration and tangential spread of migrating neurons by regulating the balance between cell adhesion and repulsion. Down syndrome cell adhesion molecules (DSCAMs), a small group of transmembrane proteins of the immunoglobulin superfamily, are also known to be involved in neuronal migration during cortical development. In their original article, Mitsogiannis et al. show that an increased dosage of DSCAMs, which is observed in human disorders, affects morphology and migration of cortical interneurons.

Extracellular signaling components, which are known as axon guidance molecules, also regulate neuronal migration. Yamagishi et al. and Gonda et al. review the involvement of two well-known axon guidance families of proteins, Netrin and Robo/Slit, respectively, in neuronal migration during neocortical development and further discuss their participation in human developmental pathologies.

The termination of neuronal migration is the final essential step for the formation of the fine laminar structure of the cerebral cortex, where neurons detach from the RG scaffold and initiate differentiation. Hatanaka and Hirata summarize the critical roles played by multiple microenvironmental factors, including the ECM, Cajal–Retzius cells, RG cells, and neighboring neurons, during the terminal phase of neuronal migration.

In addition to the signaling action of molecular factors, recent studies have begun to reveal the central importance of mechanical forces in controlling neuronal migration during cortical development. In their review, Minegishi and Inagaki discuss the relevance of forces created between neurons and their surrounding environment and those produced within cells in controlling neuronal migration and molecular mechanisms underlying these forces. Novel insights into these forces are provided by Iwashita et al. who present a novel strategy of measuring tissue stiffness using glyoxal as a fixative combined with AFM. They then used this method to investigate the link between mechanical properties and species-specific brain structures, comparing mice, chicks, turtles, and ferrets. In addition to mechanical forces, the electrical properties of migrating neurons and their cellular environments also influence neuronal migration. Medvedeva and Pierani review the fascinating mechanisms underlying the electric field-mediated control of neuronal migration and maturation.

The evolution of the mammalian cerebral cortex is considered fundamental for the acquisition of higher brain functions. Accumulating evidence indicates that the specific adaptation of progenitor cell proliferation and neuronal migration mechanisms play critical roles in neocortical evolution. Amin and Borrell review the role of ECM molecules in progenitor cell proliferation, neuronal migration, and the evolution of cortical folding. Cortay et al. provide new data from *ex vivo* analysis of the embryonic macaque cortex, demonstrating that the radial migration speed and trajectory vary significantly between cortical areas and layers in primates, exhibiting unique features in an area- and layer-specific manner.

Our Research Topic ends with a review by Hansen and Hippenmeyer. They discuss the different extracellular elements that define the microenvironment that radially migrating neurons encounter, as well as experimental assays and methods to study non-cell-autonomous mechanisms of neuronal migration and brain development.

By assembling these articles together, this special issue provides an updated picture of the field and highlights the importance of the interplay between extrinsic signals and migrating neurons during neocortical development. We are confident that this collection will be of interest to both new and established scientists and help promote research in this fascinating field.

## Author Contributions

All authors listed have made a substantial, direct and intellectual contribution to the work, and approved it for publication.

## Conflict of Interest

The authors declare that the research was conducted in the absence of any commercial or financial relationships that could be construed as a potential conflict of interest.

